# Supplementary material for: Developing a pricing model for general medical consultation services among private consulting rooms in Harare, Zimbabwe
Source: PLoS One. 2025 Dec 12;20(12):e0324572. doi: 10.1371/journal.pone.0324572 (PMC12700376; doi:10.1371/journal.pone.0324572)
Supplement: S1 Fig — This scatterplot shows the relationship between estimated profits and ideal profits (from the predictive model) for 170 general medical practices in Harare, Zimbabwe. (PDF) [file pone.0324572.s001.pdf]

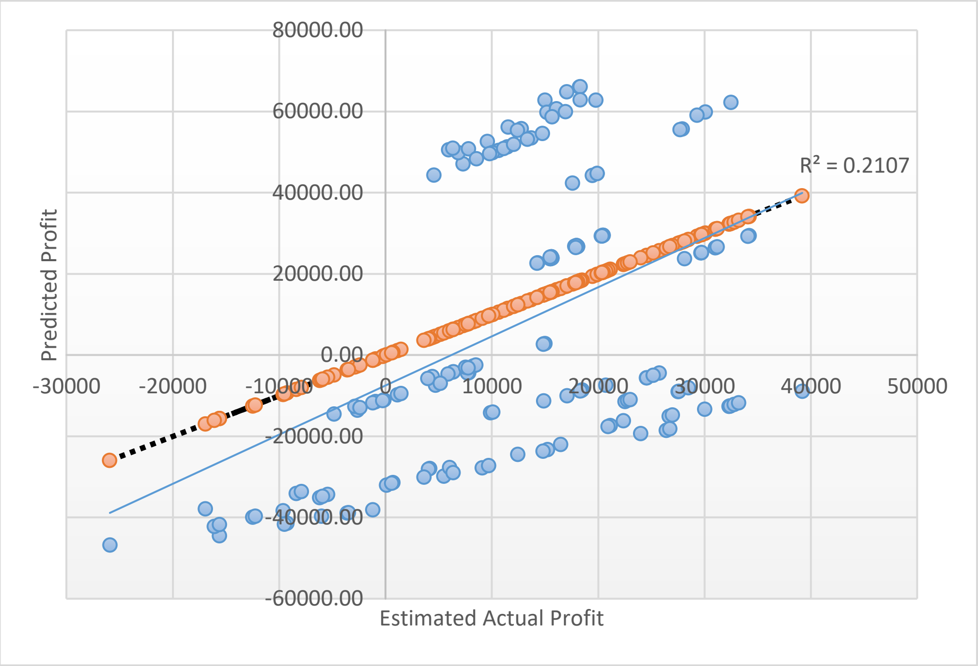

S1 Fig. Relationship between Estimated Actual Profits and Ideal Profits for General Medical Practices in Harare, Zimbabwe.

Scatterplot showing the relationship between estimated profits and ideal profits (from the predictive model) for 170 general medical practices in Harare, Zimbabwe. The broken black line with red dots represents perfect agreement (y=x), where estimated profits equal ideal profits. The R-squared value of 0.21 indicates a moderate relationship between the two.
