# Supplementary material for: Developing a pricing model for general medical consultation services among private consulting rooms in Harare, Zimbabwe
Source: PLoS One. 2025 Dec 12;20(12):e0324572. doi: 10.1371/journal.pone.0324572 (PMC12700376; doi:10.1371/journal.pone.0324572)
Supplement: S1 Table — This matrix assesses multi-collinearity among cost variables, patient volume, profit, and the outcome variable (consultation fee). (PDF) [file pone.0324572.s002.pdf]

S1 Table: Correlation matrix of the independent variables and the outcome variable.

Variables	(1)	(2)	(3)	(4)	(5)	(6)	(7)	(8)
(1) Rental	1.000							
(2) Equipment	0.878	1.000						
(3) Consumables	0.927	0.903	1.000					
(4) Salaries	0.831	0.773	0.762	1.000				
(5) Utilities	-0.804	-0.777	-0.787	-0.813	1.000			
(6) # of patients seen	-0.928	-0.877	-0.937	-0.777	0.786	1.000		
(7) Profit	-0.432	-0.506	-0.514	-0.386	0.322	0.453	1.000	
(8) Consultationfee	0.931	0.857	0.896	0.804	-0.771	-0.915	-0.203	1.000

As demonstrated in S1 Table, rental and equipment costs exhibit strong correlations (with coefficients >0.8) with other input variables, indicating multicollinearity. To maintain the statistical integrity of the regression model, these variables were excluded. However, given their essential role in clinic operations, both rental and equipment costs were incorporated into the final ideal consultation fee calculation. This dual-phase approach ensures methodological robustness while preserving real-world economic relevance.
