# Supplementary material for: Developing a pricing model for general medical consultation services among private consulting rooms in Harare, Zimbabwe
Source: PLoS One. 2025 Dec 12;20(12):e0324572. doi: 10.1371/journal.pone.0324572 (PMC12700376; doi:10.1371/journal.pone.0324572)
Supplement: S2 Table — This matrix presents the correlations among the variables retained after addressing multi-collinearity (consumables, salaries, utilities, number of patients seen, and profit), showing their relationship with the consultation fee. (PDF) [file pone.0324572.s003.pdf]

S2 Table: Correlation matrix with the five independent variables with minimal multi-collinearity

Variables	(1)	(2)	(3)	(4)	(5)	(6)
(1) Consumables	1.000					
(2) Salaries	0.762	1.000				
(3) Utilities	-0.787	-0.813	1.000			
(4) # of patients seen	-0.937	-0.777	0.786	1.000		
(5) Profit	-0.514	-0.386	0.322	0.453	1.000	
(6) Consultation fee	0.896	0.804	-0.771	-0.915	-0.203	1.000
