# Supplementary material for: Developing a pricing model for general medical consultation services among private consulting rooms in Harare, Zimbabwe
Source: PLoS One. 2025 Dec 12;20(12):e0324572. doi: 10.1371/journal.pone.0324572 (PMC12700376; doi:10.1371/journal.pone.0324572)
Supplement: S3 Table — This table compares the regression models for profitability and the one for fee determination. (PDF) [file pone.0324572.s004.pdf]

S3 Table : Comparison of Regression Models for Profitability vs. Consultation Fee Determination

Model	Dependent Variable	R^2	Interpretation
**Profit Comparison**	Actual vs. Predicted Profit	0.21	Expected due to practice-level heterogeneity.
**Fee Determination**	Consultation Fee	0.95	Strong evidence for cost-based pricing.

S3 Table above shows that the Multiple Linear Regression model for consultation fees ($R^2 = 0.95$) demonstrated strong predictive power, with consumables, salaries, utilities, patient volume, and profit as significant drivers ($p < 0.05$). In contrast, the profit comparison model ($R^2 = 0.21$) reflected inherent variability in profitability across practices, consistent with literature on clinic-level cost heterogeneity (citation has been provided in the manuscript [15]).
