# Supplementary material for: Developing a pricing model for general medical consultation services among private consulting rooms in Harare, Zimbabwe
Source: PLoS One. 2025 Dec 12;20(12):e0324572. doi: 10.1371/journal.pone.0324572 (PMC12700376; doi:10.1371/journal.pone.0324572)
Supplement: S4 Table — This table presents the number of patients seen per month, divided into three tertiles to facilitate categorical analysis. (PDF) [file pone.0324572.s005.pdf]

S4 Table: Showing the distribution of number of patients seen across the three tertiles

Three quantiles of Patients seen	Frequency	Percent	Cumulative
Low Volume	57	33.53	33.53
Medium Volume	61	35.88	69.41
High Volume	52	30.59	100
Total	170	100	

As demonstrated in S4 Table above, the number of patients seen per month was divided into three tertiles to facilitate categorical analysis. These tertiles were based on the distribution of responses, with the low volume group representing the lowest third of patient counts (1st tertile), the medium volume group covering the middle third (2nd tertile), and the high volume group comprising the highest third (3rd tertile). The cut-off values for these groups corresponded to the 33rd and 66th percentiles of the overall distribution of patients seen.
