# Supplementary material for: Developing a pricing model for general medical consultation services among private consulting rooms in Harare, Zimbabwe
Source: PLoS One. 2025 Dec 12;20(12):e0324572. doi: 10.1371/journal.pone.0324572 (PMC12700376; doi:10.1371/journal.pone.0324572)
Supplement: S1 File — This questionnaire was used to collect data from general practitioners operating private consulting rooms. (PDF) [file pone.0324572.s006.pdf]

S1 File: Data collection questionnaire for private consulting rooms in Harare

The questionnaire used for data collection is provided below. Clinics reported annualized financial data anchored to **June 2023**, the median month of the fiscal year, to standardize responses and control for seasonal variability*:* -

(NB: Tick appropriate answer and complete the questions below)

General Information

1.Are you a General Practitioner who operates a Surgery? Yes/No

2. What is your Gender? Male/Female

3. Where is your Surgery located? CBD/Low Density/High Density/Medium Density

4.How many years experience do you have practicing as a Medical Doctor? ……...

5. For how many years has your Surgery been operational? …………

Registration and Renewal Costs

6. In 2023 did you register or you were renewing? Renewing/Registering

(If you were renewing skip to question number 11)

7. How much did you pay to MDPCZ in 2023 for Registration?...

8. How much did you pay to HPAZ for Registration?.......

9. How much did you pay to City of Harare for Registration?......

10. How much did you pay to AHFoZ for registration?......

11. How much did you pay to MDPCZ for Renewal? …….

12. How much did you pay to HPAZ for Renewal? ………

13. How much did you pay to City of Harare for Renewal?..... ….

14. How much did you pay to AHFoZ for Renewal? ……..

Practice Operating Costs

15. Did you construct/build your Surgery or you are Renting? …..

16. How much did you spend on construction?........

17. How much are you paying for Rent? ………..

18. Approximately what is the size of all your working rooms (in square meters)? ………..

19. Do you have impervious floors ? Yes/No

20. Do you have a oil painted walls? Yes/No

21. Do you have a desk for the receptionist? Yes/No

22. Do you have a desk for the doctor? Yes/No

23.Do you have impervious chairs for the staff and client? Yes/No

24.How many impervious chairs do yo have? …….

25. Do you have a dressing trolley? Yes/No

26. Do you have a couch in functional order ? Yes/No

27. Do you have a foot stool in functional order? Yes/No

28. Do you have a screen? Yes/No

29. Do you have an X-ray viewing box? Yes/No

30. Do you have an Oxygen trolley? Yes/No

31. If you have an Oxygen cylinder, What is the size (in Kgs)? …….

32. Do you have a stethoscope? Yes/No

33. Do you have Reference Books? Yes/No

34. Do you have pedal lined bins ? Yes/No

35. If yes, how many pedal lined bins do you have? …………

36. Do you have fire extinguishers? Yes/No

37. If yes, how many fire extinguishers do you have? …………

38. Do you have an emergency tray with emergency drugs? Yes/No

39. Do you have a wash hand basin in the consulting room? Yes/No

40. Does your wash hand basin have splash back tiles? Yes/No

41. Besides the equipment highlighted above, what other medical equipment do you have at your Surgery?..

42.Does your wash hand basin have liquid soap? Yes/No

43. Does your wash hand basin have disposable paper towels? Yes/No

44. What was your practice's total salary cost for the month of June 2023, including the salary/remuneration of the practitioner(s) in charge/owner(s)?

Consultation Fees and Patient Volume

45. How much do you charge per patient for consultation fee?...

46. On average, how many patients do you see per month?.......

Additional Comments

47. Do you have any other comments? ………………………………………………………
