# Supplementary material for: Developing a pricing model for general medical consultation services among private consulting rooms in Harare, Zimbabwe
Source: PLoS One. 2025 Dec 12;20(12):e0324572. doi: 10.1371/journal.pone.0324572 (PMC12700376; doi:10.1371/journal.pone.0324572)
Supplement: S2 File — This document provides the official communication from the national medical regulatory body which granted permission to conduct the study among its registered practitioners. (PDF) [file pone.0324572.s007.pdf]

S2 File: Ethical Clearance Correspondence from Medical and Dental Practitioners Council of Zimbabwe

ALL CORRESPONDENCE MUST BE ADDRESSED TO THE REGISTRAR

Harare Office:
8 Harvey Brown, Milton Park
P.O Box CY 810, Causeway
Harare
Cell: 0712 544066
Tel: (04) 792195 / 793709
793707 / 790139
Email: mdpcz@mdpcz.co.zw

Bulawayo Office:
2 Robertson Street
Parkview
Bulawayo
Tel: (09) 72237/8
Cell: 0777 884 162
Website: www.mdpcz.co.zw

MEDICAL AND DENTAL PRACTITIONERS COUNCIL OF ZIMBABWE

REF/CB/fc/113/23

10 August 2023

To Whom It May Concern

Dear Sir/ Madam

RE: PERMISSION TO CONDUCT A RESEARCH AS PART OF THE REQUIREMENT TO COMPLETE MSC PUBLIC HEALTH- HEALTH ECONOMICS AND FINANCING

In reference to the above matter, the Medical and Dental Practitioners Council (MDPCZ) Education Committee agreed that Mr Chengetedzai Gota does not need Council approval to obtain information from individual practitioners. He can proceed with the research approaching individual practitioners seeking their consent.

Yours faithfully

C BENYURE (DR)
Acting Registrar

MEDICAL AND DENTAL PRACTITIONERS COUNCIL REGISTRAR
2023 -08- 10
8 Harvey Brown Ave, Milton Park, Hre
P.O. Box CY810, Causeway
Telephone: 0242 792195
