# Supplementary material for: Developing a pricing model for general medical consultation services among private consulting rooms in Harare, Zimbabwe
Source: PLoS One. 2025 Dec 12;20(12):e0324572. doi: 10.1371/journal.pone.0324572 (PMC12700376; doi:10.1371/journal.pone.0324572)
Supplement: S3 File — This document provides the formal ethical approval for the study. (PDF) [file pone.0324572.s008.pdf]

S3 File: Ethical Clearance Letter from Joint Research Ethics Committee (JREC)

Joint Research Ethics Committee

For The University of Zimbabwe,
Faculty of Medicine and Health Sciences(FMHS) &
Parirenyatwa Group of Hospitals(PGH)

UNIVERSITY OF ZIMBABWE
Faculty of Medicine
& Health Sciences

JREC Office No.4, 5th Floor, Faculty of Medicine and Health Sciences Building
Telephone: +263 242 708140/791631 Extns 2241/2242
Email: jrec.office@gmail.com - website: www.jrec.uz.ac.zw

Parirenyatwa
Group of Hospitals

APPROVAL LETTER

Date: 11 September 2023 **JREC Ref: 302/2023**

Names of Researcher **Chengetedzei Gota**

Address: **UZ – Department of Primary Health Care Sciences, Family Medicine, Global and Public Health Unit.**

RE: DEVELOPING A PRICING MODEL FOR GENERAL MEDICAL CONSULTATION SERVICES AMONG PRIVATE CONSULTING IN HARARE, ZIMBABWE.

Thank you for your application for ethical review of the above mentioned research to the Joint Research Ethics Committee. Please be advised that the Joint Research Ethics Committee has reviewed and approved your application to conduct the above named study. You are still required to obtain MRCZ and RCZ approval before you commence the study if required by the nature of your study.

- **APPROVAL NUMBER:** **JREC/302/2023**
- **APPROVAL DATE:** **11/09/2023**
- **EXPIRY DATE:** **10/09/2024**

This approval is based on the review and approval of the following documents that were submitted to the Joint Ethics Committee:

a) Completed Application Form
b) Full Study Protocol
c) Informed Consent in English and/or appropriate local language

After this date the study may only continue upon renewal. For purposes of renewal please submit a completed renewal form (obtainable from the JREC office) and the following documents before the expiry date:

a. Progress Report
b. A Summary of Adverse Events
c. A DSMB Report

- **MODIFICATIONS:**

Prior approval is required before implementing any changes in the protocol including changes in the informed consent.

- **TERMINATION OF STUDY:**

On termination of the study you are required to submit a completed request for termination form and a summary of the research findings / results.

Yours sincerely,

Dr Fiona Makoni
JREC Chairperson
FM/ub

End
