# Supplementary material for: Developing a pricing model for general medical consultation services among private consulting rooms in Harare, Zimbabwe
Source: PLoS One. 2025 Dec 12;20(12):e0324572. doi: 10.1371/journal.pone.0324572 (PMC12700376; doi:10.1371/journal.pone.0324572)
Supplement: Attachment — Submitted filename: Response to Reviewers.doc [file pone.0324572.s010.doc]

Chengetedzai Gota

19491 Mutual Park Budiriro 5B Harare | +263779724286 | chengegota@gmail.com

4 July 2025

Fatima Suleman
Academic Editor
**PLOS ONE**

Dear Fatima Suleman,

RE: RESPONSE TO REVIEWERS - MANUSCRIPT ID: PONE-D-25-17786: *Developing a Pricing Model for General Medical Consultation Services among Private Consulting Rooms in Harare, Zimbabwe*

Thank you for the opportunity to revise and resubmit our manuscript to PLOS ONE. We appreciate the constructive feedback we received from you and from the reviewers, which has significantly contributed to strengthening the manuscript. Please find below our detailed, point-by-point responses to each comment, along with references to changes made in the revised manuscript.

**Reviewer 1 Comments**

**Comment 1**: Concerns on the ability of practices to provide complete financial data.

**Response**: Thank you. We confirm that the Health Professions Authority Zimbabwe (HPAZ) database was used as the sampling frame. This database is updated annually and contains verified phone numbers, email addresses, and physical addresses, enabling reliable communication with health institutions. We used a structured, predominantly closed-ended questionnaire that facilitated ease of completion. The tool is now included as Supporting Information. The high response rate achieved in this study was also supported by ethical clearance from the Joint Research Ethics Committee of Zimbabwe (JREC) and official correspondence from the Medical and Dental Practitioners Council of Zimbabwe (MDPCZ), which clarified that no additional Council approval was required beyond individual consent. These approvals helped enable trust among participants and encouraged cooperation. Both letters are included in the Supplementary Materials for reference.

**Comment 2**: Clarification needed on sampling, response rates, and possible bias.

**Response**: The questionnaire was distributed to 180 private consulting rooms, of which 170 (94.4%) responded. This high response rate is attributable to the simplicity of the questionnaire and the structured tick-box design. The study's endorsement by the Medical and Dental Practitioners Council of Zimbabwe was also a contributing factor to the high response rate. Equipment valuation was guided by the HPAZ Inspection Manual, which outlines minimum equipment standards. We corrected the sample size reference in the manuscript (page 5).

**Comment 3**: Clarify whether costs were annual and specify the reference period.

**Response**: The study was cross-sectional. Data were collected in September 2023 but referenced to June 2023 — a median month to mitigate seasonal fluctuations. Monthly costs (rent, salaries, utilities, consultation fees) were annualized by multiplying by 12. Equipment costs were estimated based on market prices and annuitized over a five-year useful life. This has been clarified in the revised Methods section.

**Comment 4**: Justify the 20% acceptable profit margin.

**Response**: We adopted a 20% profit markup to represent a conservative estimate of sustainable practice margins, as supported by cost-plus pricing principles in health economics (citation has been provided in the manuscript on page 8). Private healthcare providers in low-middle income countries typically operate at 15-25% profit margins to sustain operations and reinvest in services. We clarified this on page 8 and added the citations.

**Comment 5**: Repetition in the participant breakdown.

**Response**: We have deleted the redundant statement in the Results section for improved clarity.

**Comment 6**: Clarify that Table 1 represents full-year cost estimates.

**Response**: We updated the title of Table 1 to specify that the values represent annual costs.

**Comment 7**: Include standard deviations in Table 2.

**Response**: Standard deviations have been included in Table 2. This helps to illustrate the variability in practice-level costs across different locations.

**Comment 8**: Concern on the exclusion of rentals in the regression model.

**Response**: A multi-collinearity check revealed high correlation (r > 0.8) between rentals and other input variables (equipment and rentals). To ensure model stability, rentals and equipment were excluded. This rationale is now clearly stated in the submitted Supplementary Materials Tables S1 and S2 which show correlation matrixes.

**Comment 9**: Elaborate on the implications of statistically significant differences in consultation fees across suburbs.

**Response**: We expanded the Results section (page 15) to explain that significant differences suggest geographic price variation, potentially reflecting cost structures and socioeconomic factors. This informs the need for differentiated pricing models.

**Comment 10**: The assumption of a 20% profit margin may not be appropriate if the doctor's financial reward comes purely from the practice profit after expenses are accounted for.

**Response**: We appreciate the reviewer's concern. In our study, salaries include the owner’s/practitioners' remuneration, which is accounted for as part of the payroll costs. The 20% profit margin is calculated on top of these costs, representing a return on investment rather than the practitioner's salary. This approach allows for a clearer distinction between operational costs, including practitioner remuneration, and the profit generated by the practice.

**Reviewer 2 Comments**

**Comment 1**: Possibility of missing cost categories.

**Response**: A thorough literature review and expert consultation guided the selection of input cost categories. These include registration fees, rent, equipment, consumables, salaries, and utilities. Pre-testing confirmed these as comprehensive.

**Comment 2**: Make the questionnaire and data available.

**Response**: Both the questionnaire and the de-identified dataset are provided as Supporting Information.

**Comment 3**: Does the model's estimated consultation fee cover operating costs?

**Response**: We have conducted an internal validation and confirm that, on average, the model-derived consultation fees cover annual operating costs. A supplementary table has been added to illustrate this comparison.

**Comment 4**: Risk of misinterpretation of the average ideal consultation fee.

**Response**: Thank you for this observation. We have made a correction in the manuscript were we had written “ideal average” to “estimated ideal average” consultation fee to reflect its indicative, not prescriptive, purpose.

**Comment 5**: Clarify whether actual profit is reported or estimated.

**Response**: Actual profit was calculated by the authors from cost and revenue data reported by the practices, not self-reported as a net profit figure.

**Comment 6**: Tables 6 and 7 need more interpretation.

**Response**: The narrative has been expanded to explain that the ANOVA and Bonferroni tests were essential to assess whether pricing variations were statistically significant across geographic strata.

**Comment 7**: Concern on why rental was excluded from the model.

**Response**: One of the assumptions for multiple linear regression analysis is that the independent variables should not be highly correlated with each other i.e there should be no multicollinearity. As seen in Table S1 in the Supplementary Materials document, there is a high correlation (magnitude of the correlation coefficients is greater than 0.8) of rental and equipment with most of the other independent variables. Thus rental and equipment were removed from the regression. The reasons for their exclusion has also been highlighted in the discussion as you have suggested.

**Comment 8**: Compare findings with WHO CHOICE data.

**Response**: We thank the reviewer for suggesting a comparison with WHO CHOICE estimates, and providing the link for accessing the site as well. As detailed in the revised Discussion (page 16), our calculated fee of **$23.25** falls within the expected range for private outpatient care in LMICs.

**Comment 9**: A data point is needed, in which for all 170 practices there is a visual representation of their operating costs, their actual profits (from their annual account), their estimated profit (from your model) and their ideal profits, also from your model.

**Response**: We appreciate the reviewer's suggestion to provide a visual representation of the data. In our Supplementary Materials document, we have provided a visual representation of the relationship between estimated actual profits and ideal profits for the 170 general medical practices in the form of a scatter plot (Figure S1). This is now included in the Supplementary Materials document. The plot shows a moderate relationship between the two, with an R-squared value of 0.21. This suggests that while our model captures some of the variability in ideal profits, there are other factors at play that are not accounted for. Although we did not have access to the clinics' financial records, we estimated actual profits based on the data collected (subtracted total annual costs from the total annual practice revenue). The scatter plot provides a clear visual representation of the relationship between estimated and ideal profits, and we believe it effectively communicates the strengths and limitations of our model.

**Comment 10:** Concern about model fit or predictive strength for actual profit.

**Response:** Thank you for your observation. The R-squared value of 0.2107 obtained during model validation indicates that approximately 21.1% of the variance in the estimated actual profit is explained by the model. This level of explanatory power is not unusual in health economics studies involving real-world data with high variability, especially in settings without standardized pricing systems (citation has been provided in the manuscript [17]). In health institutions costing studies, R² values below 0.30 are frequent due to unobserved heterogeneity in patient complexity and institutional factors.

Importantly, the model still offers practical utility by identifying key cost drivers and providing a conservative benchmark for pricing consultation services. While we acknowledge in the limitations section of our manuscript (page 19) the low R² (0.21) in the profit comparison model, our fee determination model (R² = 0.95) robustly links costs to prices, offering a policy-relevant tool. Table S3 has been provided in the Supplementary Materials showing a Comparison of Regression Models for Profitability and Consultation Fee Determination. The profit model’s low R² (0.21) reflects known challenges in predicting clinic profitability, while the fee model’s high R² (0.95) validates its use for pricing. The primary focus of the study was the development of a consultation fee determination model. The dataset has been shared for transparency.

**Conclusion**

We are grateful for the opportunity to revise our work. We believe these changes improve the rigor and clarity of our manuscript. The submission includes:

- A revised manuscript with tracked changes.
- A clean version of the revised manuscript.
- This point-by-point rebuttal letter.
- Document with Supplementary Materials, which include: questionnaire, correlation matrix, scatter plots, tables, figures and ethical clearance correspondences.
- De-identified dataset.

Please don’t hesitate to let us know if additional clarification is required.

Sincerely,

Chengetedzai Gota

(Corresponding Author)
