# Supplementary material for: Developing a pricing model for general medical consultation services among private consulting rooms in Harare, Zimbabwe
Source: PLoS One. 2025 Dec 12;20(12):e0324572. doi: 10.1371/journal.pone.0324572 (PMC12700376; doi:10.1371/journal.pone.0324572)
Supplement: Attachment — Submitted filename: Response to Reviewers_2nd Review.doc [file pone.0324572.s011.doc]

Chengetedzai Gota

19491 Mutual Park Budiriro 5B Harare | +263779724286 | chengegota@gmail.com

31 October 2025

Fatima Suleman
Academic Editor
**PLOS ONE**

Dear Fatima Suleman,

RE: RESPONSE TO REVIEWERS - MANUSCRIPT ID: PONE-D-25-17786: *Developing a Pricing Model for General Medical Consultation Services among Private Consulting Rooms in Harare, Zimbabwe*

Thank you for the opportunity to revise and resubmit our manuscript to PLOS ONE for the second time after the first review. We appreciate the constructive feedback we received from you and from the reviewers, which has significantly contributed to strengthening the manuscript. Please find below our detailed, point-by-point responses to each comment, along with references to changes made in the revised manuscript.

**Reviewer 1 Comments**

**Comment 1**: Terminology regarding “interview” versus “survey questionnaire”

**Response**: We thank the reviewer for this important clarification. We have replaced all instances of “interviewed” and “interview” throughout the manuscript (Abstract, Methods, Results and Discussion sections) with more accurate terminology such as “responded to the survey” and “data collected using a questionnaire”. This change enhances the methodological precision of our description.

**Comment 2**: Omission of consultation fee and number of patients seen in the questionnare

**Response**: Regarding the omission of consultation fee and number of patients seen in the questionnaire, we would like to clarify that these variables were indeed captured in the dataset shared with the reviewers. When compiling the supplementary document with the questionnaire, the questions on consultation fee and number of patients seen were unintentionally omitted. To provide clarity and transparency, we have updated the supplementary materials to include these questions, which were part of the original data collection process. The dataset, as shared with the reviewers, includes responses on these variables, which were utilized in our analysis.

**Comment 3**: Clarification on consumables costing methodology

**Response**: We appreciate the reviewer’s comment regarding the costing methodology in relation to the consumables. Associated consumables including masks, gloves, syringes, linen savers, cleaning materials, liquid soap, and paper towels were costed as standard inclusions. This assumption ensured consistency across practices and accounted for commonly bundled inputs that may not have been individually listed by respondents. We have now included this explanation in the Methods section of the manuscript on page 8.

**Reviewer 2 Comments**

**Comment 1**: Specification of currency in the study.

**Response**: We have now explicitly specified in the Methods section (Page 7) that all costs are reported in United States Dollars (US), anchored to June 2023 values. This clarification has also been added to the notes of Tables 1, 2, and 4.

**Comment 2**: Enhancement of Table 1

**Response**: We have improved Table 1 by adding standard deviations for all continuous variables. Additionally, we have included descriptive text below table 1 summarizing the key characteristics of the participating practices.

**Comment 3**: Clarification of the dependent variable in the regression analysis.

**Response**: We have added explicit text before Table 3 stating: “A multiple linear regression model was developed with the consultation fee as the dependent variable, to examine the influence of key cost-related factors such as consumables, salaries, utility costs, number of patients seen, and actual profit”. This provides better signposting for readers.

**Comment 4**: Clarification of asterisk on “Acceptable Profit” in Table 4

**Response**: We have removed the asterisk next to "Acceptable Profit" in Table 4. This symbol was originally included to indicate that Acceptable Profit was used to calculate the estimated ideal consultation fee. However, since the rationale for utilizing Acceptable Profit instead of Actual Profit is explained in the paragraph below the table, the asterisk is no longer necessary.

**Comment 5**: Addition of specific WHO CHOICE data in the Discussion

**Response**: As per the reviewer’s recommendation, we have incorporated specific outpatient consultation cost estimates from WHO-CHOICE into the Discussion section (Page 19). These estimates (US$ 20 – US$ 30) closely align with our study’s ideal consultation fee of US$ 23.28, providing readers with context and reinforcing the relevance of our findings within global cost-effectiveness standards.

**Comment 6**: Analysis of factors explaining variation in profit levels across practices

**Response**: We thank the reviewer for raising this important point. To systematically investigate the drivers of profit variation, we conducted additional analyses exploring the association between profit levels and two key factors: suburb density and patient volume. We created a new 'Profit Ratio' variable (Actual Profit/Acceptable Profit) to categorize practices into three financially meaningful groups. Based on this ratio, profit levels were categorised into three groups; Below Target Profit (<0.8), Within Target Profit (0.8–1.2), and Above Target Profit (>1.2). Similarly, the number of patients seen per month was divided into three tertiles to facilitate categorical analysis. The table showing the three tertiles of patient volume has been included in the Supplementary Materials (Table S1). Chi-square tests revealed statistically significant associations for both suburb density (χ²(6)=106.75, p<0.001) and patient volume (χ²(4)=83.89, p<0.001).

The results reveal a clear pattern: high-density suburban and high-volume practices are overwhelmingly represented in the 'Above Target' profit category, whereas low-density and low-volume practices are predominantly in the 'Below Target' category. We have included these findings as **Tables 8 and 9 in the manuscript**, which detail the Chi-square tests for suburb density and patient volume, respectively. These findings provide a clear, data-driven explanation for the observed profit differences. The updated de-identified dataset, which includes the new variables (Profit Ratio, Profit Category, and Patient Volume tertiles), has been attached for your reference. We have also included this important finding in the Abstract (Page 1), Discussion (Page 20) and Conclusion (Page 22) sections of our manuscript.

**Conclusion**

We are grateful for the opportunity to revise our work. We believe these changes improve the rigor and clarity of our manuscript. The submission includes:

- A revised manuscript with tracked changes.
- A clean version of the revised manuscript.
- This point-by-point rebuttal letter.
- Supplementary Materials document with updated questionnaire.
- Updated de-identified dataset.

Please don’t hesitate to let us know if additional clarification is required.

Sincerely,

Chengetedzai Gota

(Corresponding Author)
